# Suberoylanilide hydroxamic acid induces apoptosis and sub-G1 arrest of 320 HSR colon cancer cells

**DOI:** 10.1186/1423-0127-17-76

**Published:** 2010-09-17

**Authors:** Pei-Chang Sun, Ching Tzao, Ban-Hen Chen, Chen-Wei Liu, Cheng-Ping Yu, Jong-Shiaw Jin

**Affiliations:** 1 Graduate Institute of Pathology and Parasitology, Tri-Service General Hospital, National Defense Medical Center, Taiwan, R.O.C; 2 Division of Thoracic Surgery, Tri-Service General Hospital, National Defense Medical Center, Taiwan, R.O.C; 3 Department of Surgery; Graduate Institute of Medical Science; Tri-Service General Hospital, National Defense Medical Center, Taiwan, R.O.C; 4 Department of Pathology, Tri-Service General Hospital, National Defense Medical Center, Taiwan, R.O.C

## Abstract

**Background:**

Histone deacetylases and histone acetyl transferases covalently modify histone proteins, consequentially altering chromatin architecture and gene expression.

**Methods:**

The effects of suberoylanilide hydroxamic acid, a HDAC inhibitor, on 320 HSR colon cells were assessed in 320 HSR colon cancer cells.

**Results:**

Concentration and time-dependent inhibition of 320 HSR cell proliferation was observed. Treatment of 320 HSR cells with 5 μM SAHA for 72 h significantly inhibited their growth by 50% as compared to that of the control. Fluorescence-activated cell sorting analysis demonstrated significant inhibition of cell cycle progression (sub-G1 arrest) and induction of apoptosis upon various SAHA concentrations after 48 h. In addition, the anti-apoptosis proteins, survivin and Bcl-xL, were significantly inhibited by SAHA after 72 h of treatment. Immunocytochemistry analysis revealed that SAHA-resistant cells were positive for cyclin A (85%), ki-67 (100%), p53 (100%), survivin (100%), and p21 (90%) expression. Furthermore, a significant increase cyclin A-, Ki-67-, p53-, survivin-, and p21-positive cells were noted in SAHA-resistant tumor cells.

**Conclusion:**

Our results demonstrated for the first time in 320 HSR colon adenocarcinoma cells that SAHA might be considered as an adjuvant therapy for colon adenocarcinoma.

## Background

Histone deacetylases (HDAC) and histone acetyltransferase have antagonistic actions on histones, depending on the cell state [1,2]. Epigenetic regulation of gene expression has been the subject of growing interest, and HDAC inhibitors (HDACi) represent a new target for treatment of cancers [3,4]. One HDACi, suberoylanilide hydroxamic acid (SAHA), has promising anticancer activity through covalent modification of histone proteins, specifically inhibiting HDACs. A recent study reported that SAHA induced the accumulation of acetylated histones by direct interaction with HDAC [5].

Previous studies have shown that SAHA induces apoptosis through activation of the apoptotic pathway [6] and is associated with down-regulation of anti-apoptotic proteins and activation of pro-apoptotic protein expression. In addition, SAHA induces p21-mediated cell cycle arrest and cell death in cancer cells [7].

For colon cancers, surgery to remove a segment of colon tissue constitutes the principle therapy [8]. In cases with metastatic lesions or high stage disease, surgery is followed by chemotherapy to ablate any remaining cancer cells [9]. Although the prognosis of patients with colon cancer has recently improved with advanced therapy, some cases remain refractory to advanced therapy. Thus, development of new target therapies is necessary for the successful treatment of those cases.

To our best knowledge, the effects of SAHA were not studies in 320 HSR colon adenocarcinoma cells in previous publication. Here, we analyzed the effect of SAHA on a colon cancer cell line, 320HSR cells. SAHA efficiently inhibited cell growth and induced cell apoptosis, indicating that SAHA might represent a promising adjuvant therapeutic agent for the treatment of colon cancer.

## Methods

### Cell line

The human colon adenocarcinoma cell line, 320 HSR (BCRC) cells, were cultured in 90% RPMI 1640 medium supplemented with 2 mM L-glutamine adjusted to contain 1.5 g/L sodium bicarbonate, 4.5 g/L glucose, 10 mM HEPES, 1.0 mM sodium pyruvate, and 10% heat-inactivated fetal bovine serum. Cell monolayers were routinely grown to confluence at 37°C in 5% CO_2 _prior to analysis. This study has been approved by the Internal Review Board of Tri-Service General Hospital (No. 097-05-147).

### Cell proliferation analysis

For MTT (3-(4, 5-dimethylthiazol-2-yl)-2, 5-diphenyltetrazolium bromide) assays, 320 HSR cells were cultured in 96-well culture plates at a density of 7000 cells/well with 200 μL culture medium. Suberoylanilide hydroxamic acid (SAHA) was purchased from Cayman Chemical Company, Ann Arbor, Michigan, USA. After overnight plating, SAHA at concentrations of 0.5, 1, 2.5, 5, 10, 20, and 25 μM was added for 24 h, 48 h and 72 h. After culturing overnight, 20 μL MTT (5 mg/mL in PBS) was added to each well. After additional 0.5 h at 37°C, the supernatant was added to 100 μL DMSO to dissolve the blue formazan crystals produced by the mitochondrial succinate dehydrogenase of the living cells. Cell viability proportionate to optical density was measured using a colorimetric assay of mitochondria activity. Drug resistance was represented as the percentage of live cells surviving after drug treatment relative to control cells. Absorbances were measured using a spectrophotometer at a wavelength of 570 nm.

### Western blot analysis

The following antibodies and dilutions were used: mouse anti-p21 (1:1000, Oncogene Research Products, USA); rabbit anti-survivin (1:1000, R&D System, Germany); mouse anti-cleavage of poly (ADP) ribose polymerase (C-PARP; 1: 1000, Santa Cruz, CA, USA); and mouse anti-Bcl-xL (1:1000, Santa Cruz, CA, USA). Rabbit anti-mouse (1:1000, Santa Cruz, CA, USA) and swine anti-rabbit (1:1000, Santa Cruz, CA, USA) HRP-coupled secondary antibodies at a final concentration of 1 μg/mL were also used. Specific protein bands were visualized by enhanced chemiluminescence assay (Millipore Corporation, Billerica, U.S.A). All Western blots were also immunoblotted for GAPDH to demonstrate equal loading of protein samples.

### Analysis of VEGF secretion in conditioned media by enzyme-linked immunosorbent assay (ELISA)

Vascular endothelial growth factor (VEGF) protein in the conditioned media of untreated and treated cells was determined using the R&D ELISA Kits (R&D) according to the manufacturer's instructions. Optical density was determined using a microtitre plate reader at 450 nm. Results were normalized to cell number (1 × 10^5^), and a standard curve was generated by correlating the original concentration of targeted factor and the corresponding optical densities. VEGF concentration in media samples was then calculated according to the standard curve. All experiments were carried out in triplicate.

### Fluorescence-activated cell sorting analysis

After the cells were treated with different concentrations of SAHA (1, 2.5, and 5 μM) for 48 h, the apoptotic fraction was determined using the annexin V-FITC apoptosis kit (BD Biosciences, San Diego, CA) in accordance with the manufacturer's instructions. The data were analyzed with CellQuest software (Becton Dickinson). The cells were treated with Annexin-V and propidium iodine (PI) for 30 min at room temperature in dark. Annexin-V and PI fluorescences were measured using a flow cytometer (Becton Dickinson) and analyzed with CellQuest software. Viable cells were negative for both dyes, late apoptotic cells were positive for both fluorochomes, and early apoptotic cells were positive for Annexin-V but negative for PI. For each sample, 10,000 events were acquired on a logarithmic scale for both Annexin V and PI fluorescences.

For cell cycle analysis, cells were harvested 48 h after stimulation in the absence or presence of SAHA (0.1 μM to 5 μM), washed, fixed in 95% ethanol overnight at 4°C, incubated with RNase A (50 μg/mL) for 30 min at 37°C, and incubated with PI (50 μg/mL) for 30 min at 37°C. The intracellular PI fluorescence intensities of 10,000 cells were measured in each sample using a flow cytometer (Becton Dickinson, San Jose, CA).

### Immunocytochemistry analysis

Colon cancer cell lines were cultured on cover-slides in the presence or absence of SAHA (1 or 5 μM) for 48 h. The slides were incubated with the primary antibody for one h after which they were then rinsed. Staining was carried out using the streptavidin-biotin kit (Dakocytomation); the primary antibodies used were as follows: mouse monoclonal anti-p21 antibody (Oncogene Research Products, Cambridge MA; 1: 100), anti-cyclin A (Abcam; 1: 50), anti-Ki-67 (Dakocytomation; 1: 50), anti-p53 (Dakocytomation; 1: 100), and anti-survivin (Dakocytomation; 1: 100). The positive-staining cells were counted in 3 to 4 random images of high power fields (400×).

## Results

### Effects of SAHA on cell morphology and growth

**Figure 1:**
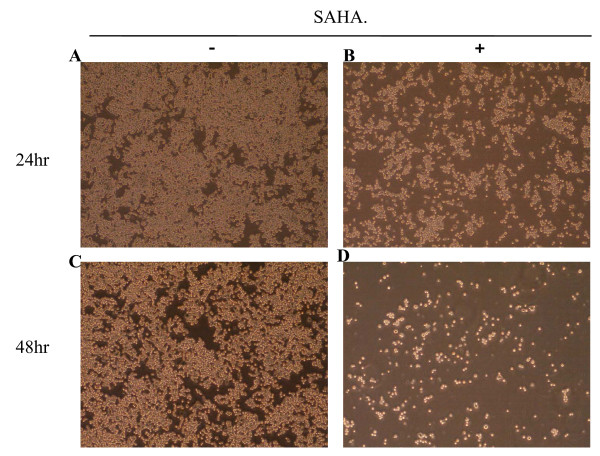
**Morphology of 320 HSR colon cancer cells after 48 h treatment with 5 μM SAHA**. Original magnification ×400.

The morphology of 320 HSR cells after SAHA treatment is shown in Figure 1. After 48 h, SAHA (5 μM) inhibited the growth of 320 HSR cells.

**Figure 2:**
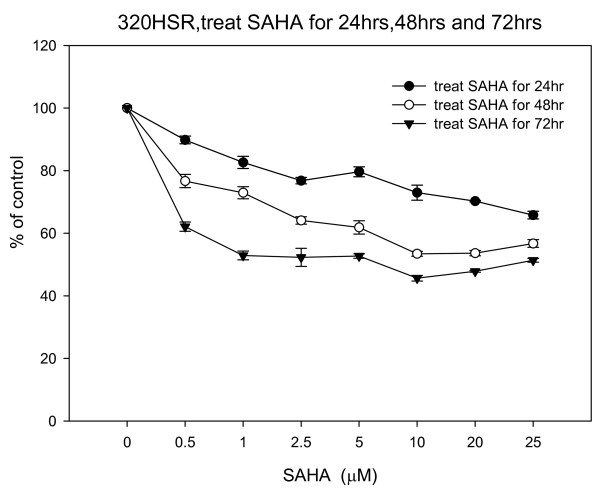
**Effects of SAHA on 320 HSR cell growth using the MTT assay**. 320 HSR cells were treated with various concentrations of SAHA for 24, 48, and 72 h.

Using the MTT assay, 320 HSR cell growth was inhibited by SAHA (Figure 2). Both dose- and time-dependent inhibitions of colon cancer cell growth were observed after 24, 48, and 72 h treatment with various concentrations of SAHA (0-25 μM). After 72 h, SAHA (5 μM) significantly inhibited 320 HSR cell growth by 50%. Concentrations of SAHA larger than 5 μM did not further inhibit cell growth.

### Fluorescence-activated cell sorting analysis

**Figure 3:**
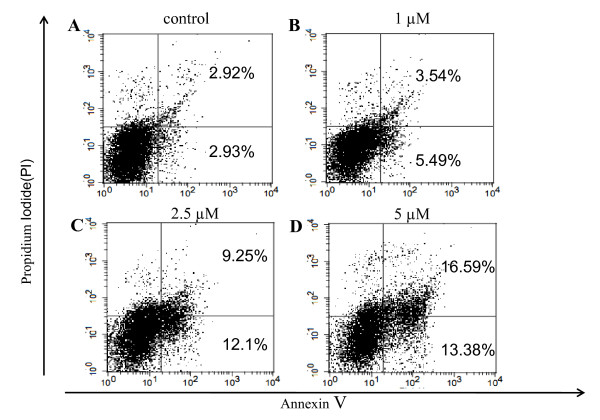
**SAHA induces apoptosis in colon cancer cell lines**. 320 HSR cells were stained with Annexin V (FITC) and propidium iodide (PI) after treatment with SAHA. Fluorescence-activated cell sorting analysis of 320 HSR cancer cell line at 48 h following treatment with 0, 1, 2.5, and 5 μM SAHA (A, B, C, D, respectively). Percentages represent Annexin V-positive/PI-negative (early apoptotic) and Annexin V-positive/PI-positive cells (apoptotic).

As shown in Figure 3, a concentration-dependent effect of SAHA in inducing apoptosis in 320 HSR cells was observed. Apoptosis of 320 HSR colon cancer cells was assessed using FACS analysis after cells were cultured with 1, 2.5, or 5 μM SAHA for 48 h. The percentage of early apoptotic cells (Annexin V-positive/PI-negative) was 2.93% for control, 5.49% for 1 μM SAHA, 12.1% for 2.5 μM SAHA, and 13.38% for 5 μM SAHA. In addition, the percentage of late apoptotic cells (Annexin V-positive/PI-positive) was 2.92% for control, 3.54% for 1 μM SAHA, 9.25% for 2.5 μM SAHA, and 16.59% for 5 μM SAHA.

**Figure 4:**
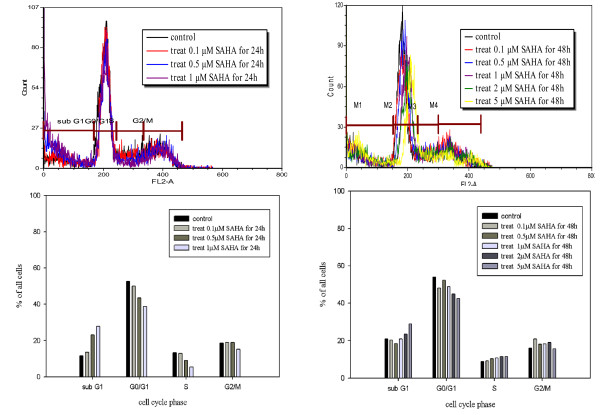
**Fluorescence activated cell sorting (FACS) analysis revealed SAHA-induced sub-G1 arrest in 320HSR cells**. Cells were harvested 48 h after stimulation in the absence or presence of SAHA (0.1 μM to 5 μM). Intracellular PI fluorescence intensities of cells are presented in the upper panels. The percentage of cells in the G0/G1 phase was significantly inhibited by SAHA treatment after 24 or 48 h. The percentage of cells in the sub-G1 phase was significantly increased in response to SAHA treatment.

For cell cycle analysis, cells were harvested 24 and 48 h after stimulation in the absence or presence of SAHA (0.1 μM to 5 μM; Figure 4). Intracellular PI fluorescence intensities are presented in the upper panels. The percentage of cells in the sub-G1 phase was significantly increased upon SAHA treatment for 24 and 48 h. In addition, the percentage of cells in the G0/G1 phase was significantly inhibited by SAHA treatment. These data suggest that SAHA induced sub-G1 arrest in 320 HSR cells.

### Effect of SAHA on expression of proteins

**Figure 5:**
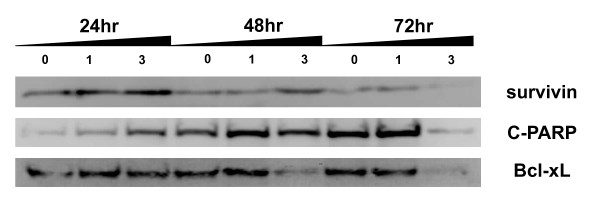
**SAHA alters survivin, C-PARP, and Bcl-xL protein levels in 320 HSR cells**. Western blot analysis of cells treated with or without SAHA (1 or 3 μM) for 24, 48, and 72 h.

**Figure 6:**
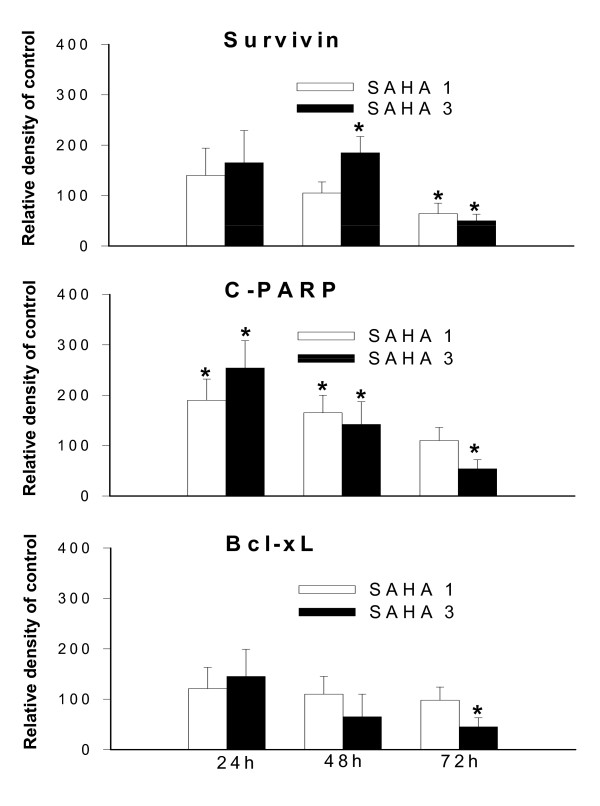
**Histogram values are means and standard error of band densitometry data measured in figure 5**. *P <0.05 vs. control.

The effect of SAHA on expression of proteins related to apoptosis and cell cycle regulation is displayed in Figure 5 and 6. Treatment of 320 HSR cells with 3 μM SAHA for 72 h significantly reduced the expression of the anti-apoptotic proteins, survivin and Bcl-xL. In addition, increased cleavage of PARP protein (c-PARP) was seen after a 72 h exposure to 3 μM SAHA.

### Effects of SAHA on VEGF concentration

**Figure 7:**
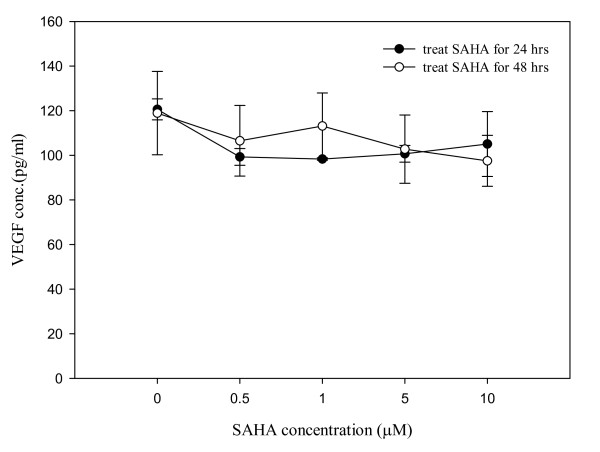
**Effects of SAHA on VEGF secretion by 320 HSR cells**. Cells were treated with different SAHA concentrations for 24 h and 48 h. VEGF concentrations in the conditioned medium were determined by ELISA and normalized to 1 × 10^5 ^viable cells.

The effect of SAHA on VEGF secretion by 320 HSR cells into the culture medium was analyzed (Figure 7). Treatment of 320 HSR cells with various SAHA concentrations (0.5 to 10 μM) for 24 and 48 h did not significantly alter the concentration of VEGF within the culture medium. VEGF concentrations were normalized to 1 × 10^5 ^viable cells.

### Immunocytochemistry analysis

**Figure 8:**
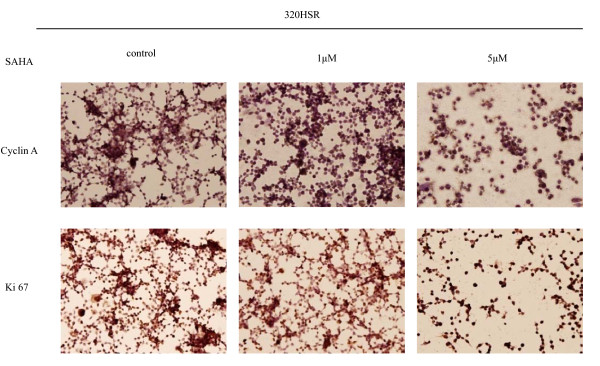
**Immunocytochemical analysis of cyclin A and Ki-67 expression in 320 HSR cells**. Cells were treated with or without SAHA (1 or 5 μM) for 48 h. Original magnification ×400.

**Figure 9:**
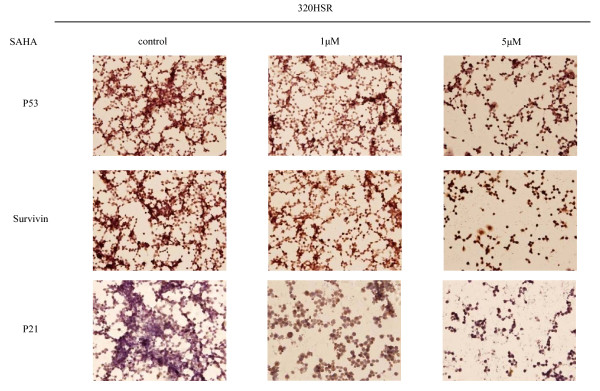
**Immunocytochemical analysis of p53, survivin, and p21 expression in 320 HSR cells**. Cells were treated with or without SAHA (1 or 5 μM) for 48 h. Original magnification ×400.

To characterize the effects of SAHA on cell cycle and proliferation proteins, alcohol-fixed cells were immunostained for cyclin A, Ki-67 (proliferation marker), p53, survivin (anti-apoptosis marker), and p21 (cyclin-dependent kinase inhibitor, Figure 8 and Figure 9). In untreated cells, the proportion of cells positive for cyclin A was 35%, Ki-67 was 80%, p53 was 90%, survivin was 70%, and p21 was 30%. After treatment of 320 HSR cells with SAHA (5 μM) for 48 h, percentage of SAHA-resistant tumor cells that were positive for cyclin A, Ki-67, p53, survivin, and p21 were 85, 100, 100, 100, and 90%, respectively. A significant increase cyclin A-, Ki-67-, p53-, survivin-, and p21-positive cells were noted in SAHA-resistant tumor cells.

Survivin expression was detected in both the nucleus and cytoplasm of 320 HSR colon cancer cells (Figure 9). Increased survivin expression was observed in SAHA-resistant cells, suggesting that survivin could counteract the growth inhibitory effects of SAHA.

With respect to the cyclin-dependent kinase inhibitor, p21, a direct target of SAHA, few positively-stained cells were observed in untreated colon cancer cells (Figure 9). After SAHA treatment, increased p21-positive cells were observed in SAHA-resistant cells, displaying both nuclear and cytoplasmic staining.

Taken together, these results indicate that SAHA inhibit the proliferation of 320 HSR colon cancer cells through induction of apoptosis and sub-G1 arrest in a concentration- and time-dependent manners. SAHA-resistant cancer cells express high levels of cyclin A, Ki67, p53, survivin, and p21.

## Discussion

HDACi inhibits acetylation of histones H3 and H4 and induce tumor apoptosis in various types of cancers [3,10,11]. The present study demonstrates for the first time in 320 HSR colon adenocarcinoma cells that SAHA has profound antigrowth activity at micro-molar concentrations. Specifically, SAHA induced apoptosis and sub-G1 arrest in 320 HSR colon cancer cells by inhibiting the protein expression of anti-apoptosis proteins, survivin and Bcl-xL.

Multiple mechanisms have been proposed to describe the effects of SAHA in different cancers [7]. Previous studies indicate that SAHA down-regulates certain anti-apoptosis proteins such as Bcl-2 and Bcl-xL [12] and up-regulates pro-apoptotic protein expression [13]. In addition, SAHA induced p21-mediated inhibition of cell cycle progression and cell death in cancer cells [7]. Although studies have suggested that SAHA-mediated apoptosis was dependent on upregulation of p21, other studies have reported that p21 expression inhibited cell death induced by HDACi [14-16]. In our study, immunocytochemistry analysis revealed increased p21-positive cells in SAHA-resistant cells, suggesting an anti-apoptotic function for p21 as well as protection from the cytotoxic effects of SAHA.

Fifty-percent inhibition of 320 HSR cell growth was observed upon 5 μM SAHA treatment. Previous clinical studies have shown that these levels can be achieved in individuals receiving the drug [17]. Studies in humans have found that SAHA induced minor side effects [17], suggesting that SAHA use as an adjuvant target therapy may be beneficial.

The antiproliferative effects of SAHA have been studied in a thyroid cancer cell line [6], human lymphoma cells [18], breast cancer [19] and non-small cell lung carcinoma [20], as well as endometrial and ovarian cancer cells [21,22]. These studies highlight that the inhibitory activity of SAHA on cancer cell growth spans many tissue types, suggesting it can be a useful agent for the treatment of a wide variety of malignancies.

Our studies using colon cancer cell lines revealed that SAHA induced apoptosis, and inhibited tumor cell growth *in vitro*. These changes were associated with down-regulation of survivin, and Bcl-xL. Each of these actions is consistent with induction of apoptosis by SAHA. Our results are consistent with a previous study using thyroid cancer cell lines; SAHA down-regulated expression of anti-apoptotic genes, including Bcl-2 and survivin, and cleavage of PARP [6,7].

Previous studies have demonstrated that survivin is a bifunctional protein that regulates cell cycle progression in mitosis as a passenger protein and blocks apoptotic pathways [5]. SAHA-induced mitotic defects can be mediated by modulation of survivin [7,23,24]. In the present study, down-regulation of survivin protein expression by SAHA contributes to the pro-apoptotic effects of SAHA.

Using immunocytochemistry analysis, SAHA-resistant tumor cells displayed a significant increase cyclin A, Ki-67, p53, survivin, and p21 expression after 48 h. Escape from SAHA-mediated apoptosis by tumor cells is through upregulation of cyclin A, Ki-67, p53, survivin, and p21 expression; therefore, combination therapies of SAHA with other pharmacological agents that target different pathways may be effective for SAHA-resistant cells.

## Conclusions

We demonstrated first time in 320 HSR colon adenocarcinoma cells that SAHA inhibited the proliferation of colon cancer cells through inducing apoptosis and sub-G1 arrest. Thus, SAHA might be considered as a potential adjuvant target therapy for colon adenocarcinoma.

## List of abbreviations

SAHA: suberoylanilide hydroxamic acid; HDAC: Histone deacetylases; MTT assay: (3-(4, 5-dimethylthiazol-2-yl)-2, 5-diphenyltetrazolium bromide) assay; PBS: phosphate buffer solution; DMSO: dimethyl sulfoxide; C-PARP: cleavage of poly (ADP) ribose polymerase; GAPDH: Glyceraldehyde 3-phosphate dehydrogenase; VEGF: vascular endothelial growth factor; ELISA: Enzyme-linked immunosorbent assay; PI: propidium iodine.

## Competing interests

The authors declare that they have no competing interests.

## Authors' contributions

PCS and CT carried out the experiments and drafted the manuscript. BHC, CWL, and CPY participated in the design of the study and performed the analysis. JSJ conceived of the study, and participated in its design and coordination and helped to draft the manuscript. All authors read and approved the final manuscript.
